# Supplementary material for: Mechanical Egg Activation and Rearing of First Instar Larvae of Sirex noctilio (Hymenoptera: Siricidae)
Source: Insects. 2023 Dec 7;14(12):931. doi: 10.3390/insects14120931 (PMC10744079; doi:10.3390/insects14120931)
Supplement: Supplementary file 1 [file insects-14-00931-s001.zip › insects-2705308-supplementary.pdf]

SUPPLEMENTARY MATERIAL

Table S1. *Pinus* sp. log batch information

Batch no.	Province	Area	*Pinus* sp.	*Pinus* sp. age (years)	Emergence season
1	Western Cape	Riversdale	*Pinus radiata*	28	Mar-Apr 2021
2	Mpumalanga	Sabie	*Pinus patula*	12-16	Oct-Nov 2022
3	Western Cape	Riversdale	*Pinus radiata*	24	Feb-Mar 2023

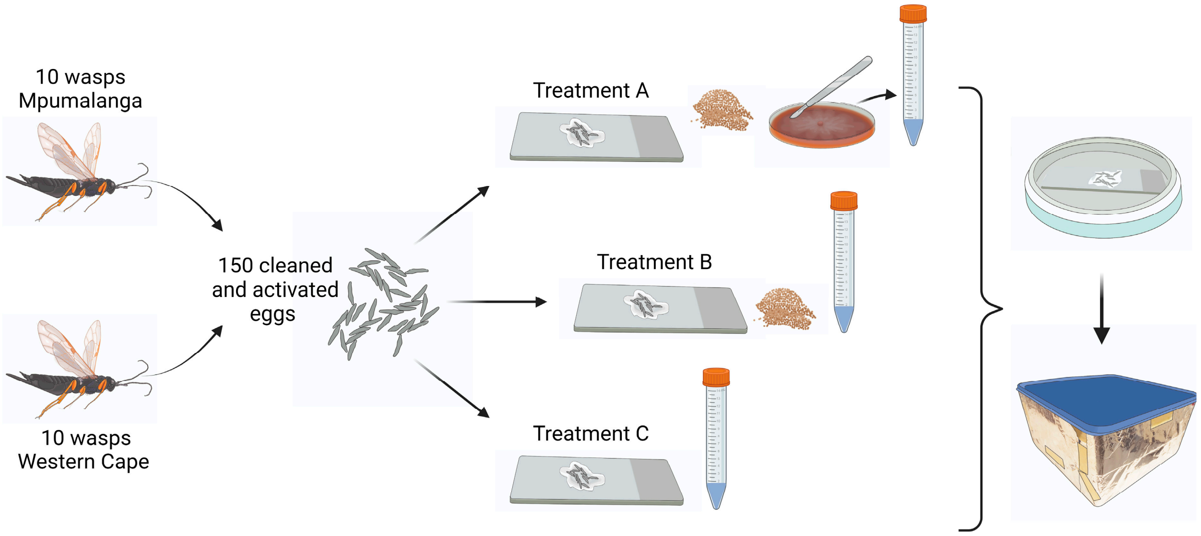

Figure S1. Treatments applied after egg activation of 10 *Sirex noctilio* female wasps from Mpumalanga and Western Cape. Each treatment contained 50 cleaned and activated eggs which was stored on a microscope slide inside a Petri dish with damp filter paper. Petri dishes were placed in a cake saver, covered in tinfoil, inside a dark incubator. (Illustration created in BioRender.com. Illustration components created by Glenda Brits, Senior Graphic Designer, Department for Education Innovation, University of Pretoria, South Africa).

Table S2. Ingredients for *Sirex noctilio* artificial diet with manufacturers

Ingredient	Manufacturer	Amount
Distilled water		257.81 ml
Agar	Sigma-Aldrich (A7921-100G)	8.20 g
**Group 1**		
Raw wheat germ	Sigma-Aldrich (W0125)	19.92 g
Torula yeast powder	Sigma-Aldrich (51475-500G)	10.55 g
**Group 2**		
Wesson salt mixture	MP Biomedicals (CAT NO. 902851)	1.05 g
Sorbic acid	Natural Resources Canada	0.73 g
Methyl paraben	Natural Resources Canada	0.73 g
Sucrose (or household sugar)	Natural Resources Canada	5.86 g
Casein from bovine milk	Sigma-Aldrich (C6554-500G)	3.52 g
Sodium propionate	Natural Resources Canada	0.45 g
**Group 3**		
Cholesterol	Sigma-Aldrich (C8667-5G)	0.35 g
Autoclaved wheatgerm oil	Essentially Natural	1.64 ml
**Group 4**		
Choline chloride	Sigma-Aldrich (C7527-100G)	0.09 g
Vanderzant vitamin mixture for insects	Sigma-Aldrich (V1007-100G)	1.55 g
Vitamin A beadlets	Natural Resources Canada	0.12 g
**Group 5**		
Alpha cellulose	Sigma-Aldrich (C6429-500G)	45.70 g
**Group 6**		
Streptomycin sulfate salt	Sigma-Aldrich (S9137)	0.3 g
SABAX pour water	Adcock Ingram	2 ml
**Group 7**		
Autoclaved pine sawdust		50 g

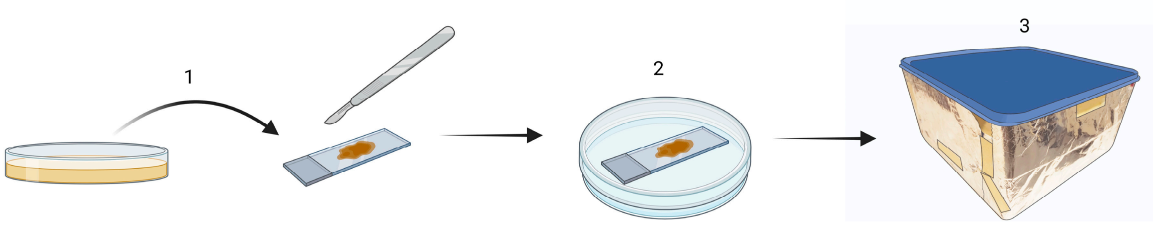

Figure S2. Rearing of *Sirex noctilio* larvae emerged from activated eggs. (**1**) A small amount of diet was placed on a microscope slide and groves were made on the diet surface using a scalpel. (**2**) The slide was placed in a Petri dish with damp filter paper and larvae were transferred onto the diet whereafter the Petri dish was sealed with Parafilm. (**3**) Petri dishes were stored in a cake saver covered in tinfoil inside a dark incubator. (Illustration created in BioRender.com. Illustration components created by Glenda Brits, Senior Graphic Designer, Department for Education Innovation, University of Pretoria, South Africa).
